# Molecular Orientation in a Variable-Focus Liquid Crystal Lens Induced by Ultrasound Vibration

**DOI:** 10.1038/s41598-020-62481-2

**Published:** 2020-04-10

**Authors:** Yuki Harada, Daisuke Koyama, Marina Fukui, Akira Emoto, Kentaro Nakamura, Mami Matsukawa

**Affiliations:** 1 Faculty of Life and Medical Sciences, Doshisha University, Kyoto, 610-0321 Japan; 2 Faculty of Science and Engineering, Doshisha University, Kyoto, 610-0321 Japan; 3 Institute of Post-LED Photonics, Tokushima University, Tokushima, 770-8506 Japan; 4 Laboratory for Future Interdisciplinary Research of Science and Technology, Tokyo Institute of Technology, Tokyo, 4259-R2-26 Japan

**Keywords:** Optical sensors, Liquid crystals

## Abstract

A method to estimate orientation direction of liquid crystal molecules three-dimensionally under ultrasound excitation was proposed and the relationship between the ultrasound vibration and the molecular orientation was discussed. Our group have reported a technique to control orientation direction of liquid crystal molecules using ultrasound vibration which could be applied to an optical variable-focus liquid crystal lens. The lens consisted of a liquid crystal layer sandwiched by two glass circular discs and a piezoelectric ring. Ultrasound vibration induces change in the refractive index of the lens, enabling the variable-focus function. The three-dimensional orientation direction of the liquid crystal molecules in the lens was predicted from the transmitted light distributions under the crossed Nicol conditions. The liquid crystal molecules were inclined from vertical alignment by the ultrasound vibration, and larger ultrasound vibration gave larger inclination of the molecules. There was a strong correlation between the distributions of ultrasound vibration and the liquid crystal molecular orientation; the molecular orientation was changed remarkably between the antinodal and nodal parts of the ultrasound flexural vibration on the glass plate and the molecules aligned towards the antinode.

## Introduction

Camera modules are inserted into electronic devices such as smartphones that are widely used in everyday life. In general, it is possible to capture images by controlling several glass or plastic optical lenses and the complex actuator system inside these camera modules. However, it is necessary to focus constantly on the object of interest when attempting to acquire images of an object that is moving in the axial direction at high speed^1^. Therefore, it is essential to improve the response speed of the optical system for this task because the lenses must be moved continuously and at high speeds in the axial direction using the actuator and gearing system^2^. In addition, portable electronic devices equipped with such camera modules may be upsized and become sensitive to shock because they contain an operating mechanism that includes elements such as lenses, actuators, and a gearing system^3^. Therefore, the design of camera modules for next-generation electronic devices must meet demands for both high response speeds and device size miniaturization. To solve these problems, our research group has developed liquid lenses and gel lenses that contain no mechanical moving parts based on use of the acoustic radiation force^4–8^. At present, we are working on optical lenses that combine liquid crystals with ultrasound techniques.

Liquid crystals are in a material state that lies between the crystalline solid and liquid states in terms of symmetry, energy, and properties. Liquid crystal molecules have various shapes, but most of them are rod-shaped and are categorized as either thermotropic or lyotropic. In addition, thermotropic liquid crystals have several subphases, including the nematic, smectic and cholesteric phases, and liquid crystal phase transitions occur depending on temperature differences, as the name thermotropic suggests. Among these subphases, nematic liquid crystals have particularly high liquidity and it is easy to control their molecular orientations using external forces such as electric and magnetic fields. This feature has allowed these materials to be widely used in optical devices such as liquid crystal displays^9–11^. As an optical liquid crystal device application, Sato and colleagues developed a variable focus liquid crystal lens using the birefringence of a nematic liquid crystal^12–16^. Although these liquid crystal lenses require complex indium tin oxide (ITO) electrode patterns to control the molecular orientations of their liquid crystals^17^, the lenses have a structure that does not require any mechanical moving parts, thus enabling device downsizing and providing high robustness for use in camera modules in electronic devices. However, it is essential to fabricate liquid crystal optical devices using transparent electrodes to apply electric fields to the liquid crystal layer. While ITO with its high electrical conductivity and transparency is in widespread general use as a transparent electrode material, ITO contains indium, which is a rare metal, and there are numerous additional problems with the use of this material, such as the large amounts of equipment and time required for the sputter deposition process^18^, the material’s low bending resistance and the trade-off relationship between resistivity and transparency that exists on the long wavelength side^19,20^. Recently, flexible liquid crystal optical devices have been investigated by several researchers^21–23^. However, inorganic materials such as ITO have limitations when forming electrodes on plastic resin films, which include the requirement for low temperature conditions and the need for the material to have bending properties similar to that of paper. For these reasons, development of liquid crystal devices without use of ITO electrodes is highly desirable. Several research groups have reported the effects of ultrasound wave propagation on nematic liquid crystals^24–26^. As a result, our research group developed a method to control the molecular orientation of these liquid crystals by applying optical device technology and devices based on use of the acoustic radiation force which have been cultivated to date^27,28^. We therefore believe that this method for control of liquid crystal molecules using ultrasound is promising as a candidate solution to the problems with the use of ITO electrodes. In a previous work, we fabricated a liquid crystal lens using this technique without using ITO electrodes and a variable-focus function was produced in this ultrasound liquid crystal lens by simply varying the sound pressure acting on the liquid crystal layer^29^.

Evaluation of the liquid crystal orientation is important in the design of liquid crystal devices. The molecular orientation of a liquid crystal in its initial state under zero external force is largely dependent on the boundary conditions at the interface between the alignment film and the liquid crystal, which is inclined at a specific angle (the tilt bias angle). Changes in the molecular orientations of liquid crystals under a static electric field can be calculated and predicted from the distribution of the electric potential. Sheffer *et al*. proposed the “crystal rotation method” to measure the tilt bias angle with high accuracy in a short time^30^. The birefringence distribution of a liquid crystal layer in its thickness direction can be observed by birefringence scanning near-field optical microscopy, which enables measurement of both the molecular orientation at the interface^31^ and the response to application of an external electric potential with measurement resolution on the scale of hundreds of nanometers^32,33^. However, to the best of our knowledge, the relationship between the three-dimensional molecular orientation of a liquid crystal and ultrasound vibration has not been investigated to date. In our previous work, it was considered that the variable focus function was produced by refractive index changes in the liquid crystal due to the ultrasound vibration. However, the relationship between the molecular orientation of the liquid crystal and the change in the optical focus has not been clarified. Furthermore, while the liquid crystal orientation in liquid crystal optical devices has been investigated previously^24–26,29^, the inclination of the molecular orientation of the liquid crystal in the thickness direction was not considered in these studies. In this work, we have evaluated both the two-dimensional orientation direction of the liquid crystal molecules and the three-dimensional orientation of these molecules, and the relationship between orientation direction and ultrasound vibration in a liquid crystal lens under ultrasound excitation has been clarified.

## Materials and Methods

**Figure 1:**
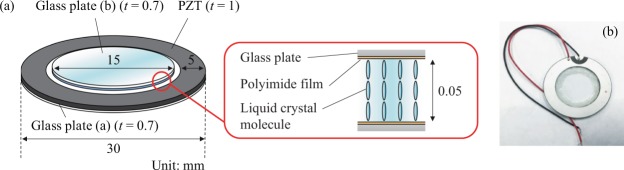
Configuration of the ultrasound liquid crystal lens.

**Figure 2:**
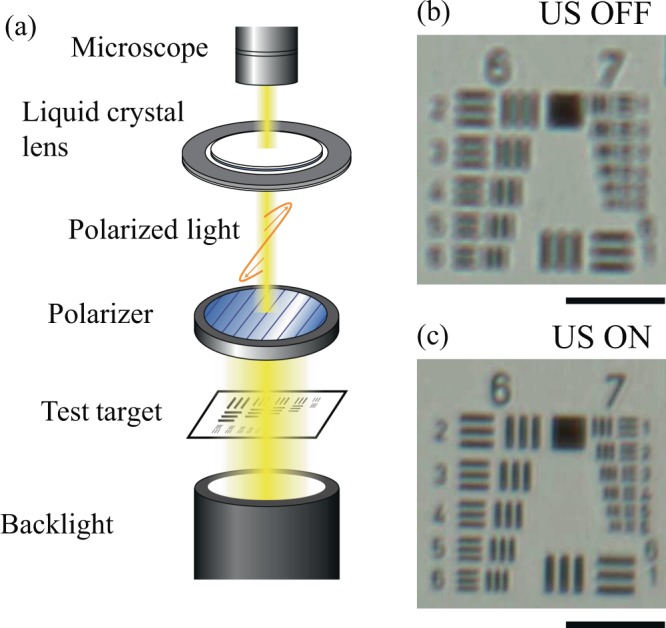
(**a**) Observational setup and (**b**) the optical images captured using the ultrasound liquid crystal lens without and (**c**) with ultrasound (US) excitation. The bars in the images represent 100 *µ*m.

The orientations of the liquid crystal molecules were evaluated using the ultrasound variable focus liquid crystal lens that is shown in Fig. 1. The nematic liquid crystal RDP85475 (DIC, Japan; refractive index *n*_0_ at 589 nm: 1.525; birefringence *Δn* at 589 nm: 0.298; transition temperature of smectic-to- nematic (SN) transition point: −10 °C; nematic-to-isotropic (NI) transition point: 123.7 °C; viscosity: 93.7 mPas) was used as the liquid crystal material. An annular piezoelectric lead zirconate titanate (PZT) ultrasound transducer (C-213, Fuji Ceramics, Japan; inner diameter: 20 mm; outer diameter: 30 mm; thickness: 1 mm that was polarized in the thickness direction was bonded to a transparent circular glass substrate (labelled plate (a); diameter: 30 mm; thickness: 0.7 mm) using epoxy. A second circular glass substrate (labelled (b); diameter: 15 mm; thickness: 0.7 mm) was bonded at the center of glass substrate (a) through a 50-*µ*m-thick silicone film to allow formation of a liquid crystal layer between the two glass substrates. The nematic liquid crystal was then injected into the gap between the two glass discs via the capillary effect and the liquid crystal layer was sealed perfectly using the epoxy. Polyimide films (vertical alignment type, SE-5811, Nissan Chemical, Japan) were formed on the inner surface of the glass substrates without rubbing, which meant that the liquid crystal molecules were oriented vertically with respect to the glass substrates because of the chemical interactions between the liquid crystal molecules and the polyimide films. The configurations of the transducer and the glass substrates were determined via finite element analysis (FEA) using the commercial FEA software ANSYS 11.0 (ANSYS. Inc., PA) to determine the resonance flexural vibration modes on the glass substrates. Excitation of the transducer using a continuous sinusoidal electric signal allowed several flexural vibration modes to be generated on the liquid crystal layer through the two glass substrates at the resonance frequencies of the entire liquid crystal lens above 20 kHz. The entire lens vibrated as a continuum body since the wavelength of the ultrasound was much larger than the thickness of the liquid crystal layer. Because of the differences in acoustic impedance among the surrounding air, the liquid crystal layer, and the glass substrate, the acoustic waves generated by inverse piezoelectric effect on the PZT ring were partially reflected at these boundaries and differences in acoustic energy density appeared between these media. As a result of this energy difference, static pressure, i.e., the acoustic radiation force^7,34–36^, acted on the liquid crystal layer from the upper and bottom sides and caused the orientation of the liquid crystal molecules to change statically. Since liquid crystal molecules have optically uniaxial anisotropy, the effective optical refractive index distribution changes along with the molecular orientation distribution, resulting in deflection of the transmitted light. The acoustic radiation force is proportional to the square of sound pressure amplitude. The axisymmetric resonance acoustic field generated in a thin circular layer (in this case, the liquid crystal layer) can be expressed as a Bessel function with concentric nodal circles and determined by the boundary condition of the sound pressure amplitude at the edge of disc^37^, inducing the axisymmetric orientation of the liquid crystal molecules. Therefore, it is possible to control both the spatial distribution of the liquid crystal molecular orientation in the liquid crystal device and its optical anisotropy by varying the resonance vibration mode and the vibrational amplitude of the glass substrate. In our previous work^29^, it was confirmed that the focal point changed along the optical axis of the lens with excitation by ultrasound vibration under the condition using one Nicol element and an optical microscope and the lens thus acted as a variable-focus lens (see Fig. 2). The focal length of this lens was dependent on the electric power consumption of the ultrasound transducer and could be controlled via the input voltage, where higher electrical consumption gave larger change in the focal length.

**Figure 3:**
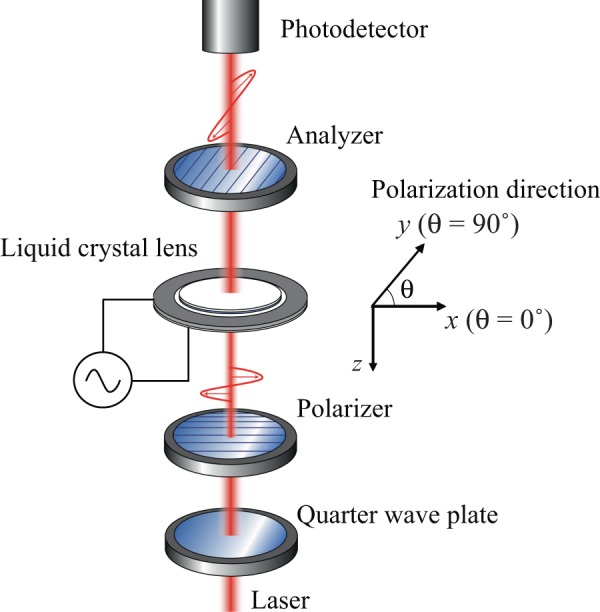
Experimental setup used for optical evaluation of the liquid crystal lens.

Figure 3 shows a schematic of the experimental system. The liquid crystal lens was positioned in parallel between a polarizer and an analyzer that were arranged orthogonally to each other (i.e., crossed Nicols conditions). A linearly polarized laser beam (He-Ne laser; *λ* = 632.8 nm; beam diameter: 2 mm) was converted into a circularly polarized beam using a quarter-wave plate. The light transmitted through the polarizer, the liquid crystal lens, and the analyzer was measured using a photodetector (PD, 2051-FS, Newport, CA). To evaluate the molecular orientation of the liquid crystal, the transmitted light distribution through the lens was measured by scanning the laser beam and rotating two polarizing plates in the in-plane direction (i.e., the incident polarization direction *θ*) while maintaining the crossed Nicols condition.

## Results and Discussion

**Figure 4:**
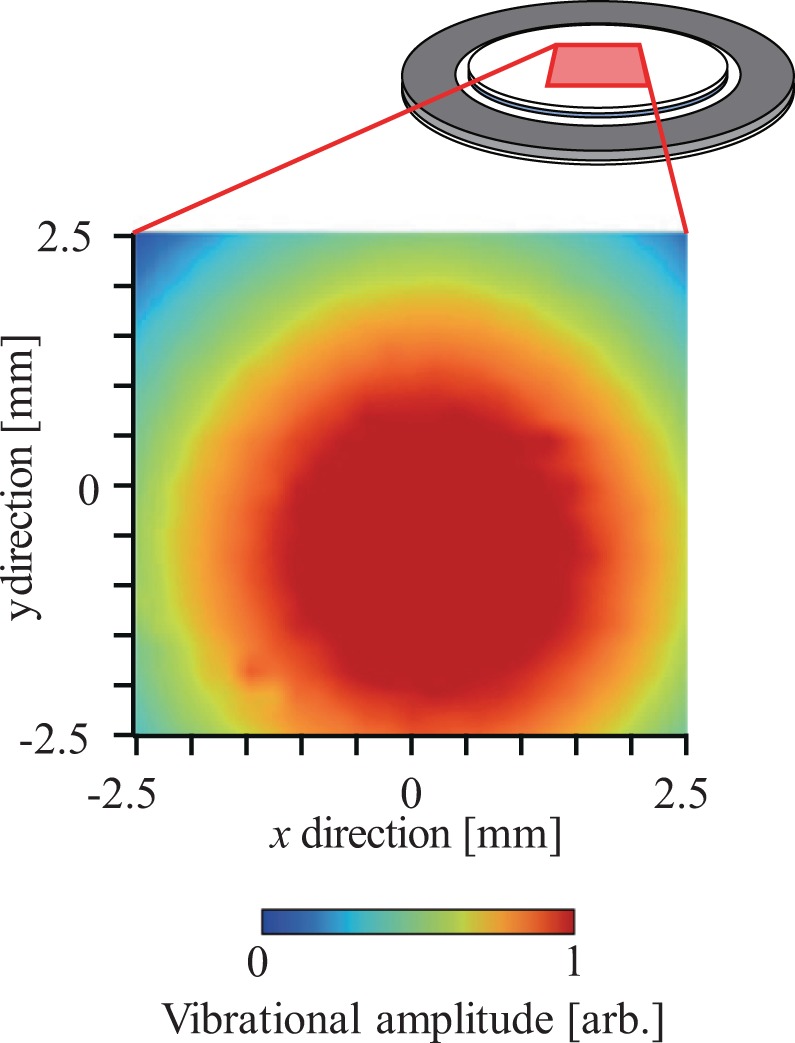
Out-of-plane vibrational displacement amplitude distribution of the liquid crystal lens at 64.2 kHz as measured using an LDV.

**Figure 5:**
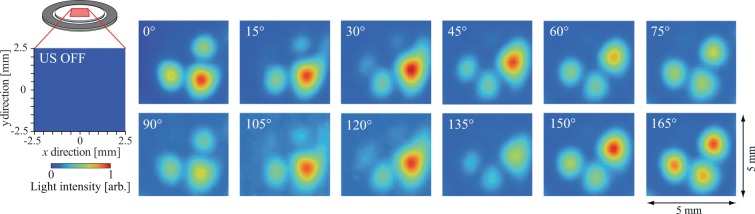
Transmitted light intensity distributions through the liquid crystal lens without and with the ultrasound excitation at 64.2 kHz.

**Figure 6:**
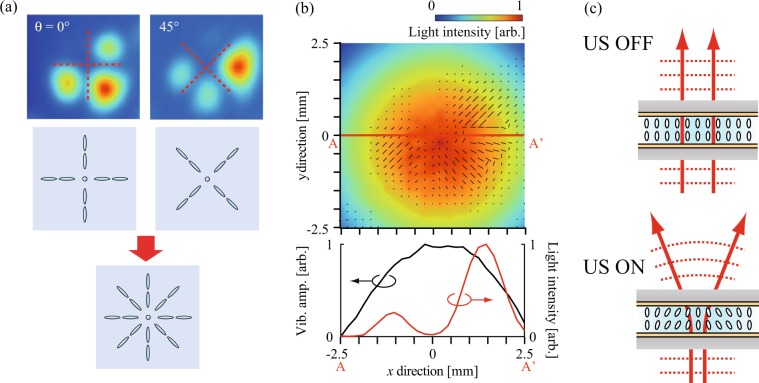
(**a**) Relationship between the transmitted light distribution and the orientation direction of the liquid crystal molecules, and (**b**) vibrational displacement amplitude (color map) and the orientation direction (bar) distributions. (**c**) Schematics of the changes in the molecular orientation and the wavefront without and with ultrasound excitation.

Figure 4 shows the measurement results for the out-of-plane vibrational distribution generated on the surface of glass plate (a) and measured using a laser Doppler vibrometer (LDV, NLV-2500, PI Polytec, Germany). The measurement area was the 5 × 5 mm^2^ area at the center of the liquid crystal lens. A concentric flexural vibration mode with two nodal circles was generated at a resonance frequency of 64.2 kHz and the center of the lens corresponded to the antinodal position with the maximum displacement of this vibration. The maximum vibrational displacement amplitude was 262 nm in the case where the input voltage was 3.5 V_pp_. Figure 5 shows the transmitted light distribution through the liquid crystal lens without and with the ultrasound excitation at an input voltage of 3.5 V_pp_ when the polarization direction of the incident light *θ* was changed from 0° to 165°. The incident light was scanned within the same 5 × 5 mm^2^ measurement area at the center of the lens used to give the results in Fig. 4, and the transmitted light intensities were normalized with respect to the maximum value at *θ* = 30°. The result in the case without ultrasound excitation (denoted by US OFF) where the transmitted light intensity in the measurement area was extremely small indicates that the molecular orientation of the liquid crystal was aligned in the vertical direction under the initial conditions because the linearly polarized light did not pass through the analyzer under the crossed Nicols conditions. When the lens was subjected to ultrasound excitation (*θ*= 0° to 165°), the transmitted light intensity increased dramatically and four peaks appeared in the circumferential direction every 90° (i.e., a cross-shaped shadow appeared). Strictly speaking, the transmitted light distributions were not perfectly axisymmetric and the intensity at one of four peaks (upper left) was lower than that at the other peaks because of the precision of our lens fabrication process (the liquid crystal was injected into the small gap at atmospheric pressure, not under vacuum). This transmitted light pattern rotated with the incident polarization direction *θ* and the same transmitted light distribution was observed every 90° for *θ*, although the maximum signal intensity differed (for example, the results for *θ* = 60° and 150° were almost identical). At the positions at which the transmitted light intensity increased greatly, the linearly polarized light that was incident on the lens was polarized elliptically through the liquid crystal layer and could then pass through the analyzer. The results shown in Fig. 5 indicate that the orientation of the liquid crystal molecules is not twisted in the thickness direction of the liquid crystal layer (if the molecular orientation is twisted in the thickness direction, two transmitted light distributions with a 90° difference are different^28^). In addition, the difference in transmitted light intensity between two cases with a 90° difference in their incident polarization directions at the same position is dependent on the birefringence of the liquid crystal molecules used. It should also be noted that the light transmitted at the center of the lens (the cross point) was constantly small at every incident polarization direction and the cross-shaped shadow directions corresponded with the incident polarization directions *θ* and *θ* + 90° (see Fig. 6(a)). These results indicate that the molecular orientation of the liquid crystal was inclined toward the center of the lens axisymmetrically with the vibration antinode and the vertical orientation was maintained at the center because the incident linearly polarized light at the center was not polarized elliptically. This is because the acoustic radiation force can be expressed as a function of the spatial gradient of the sound pressure amplitude, and the force does not act at the antinodal position of the sound pressure (at the central part) theoretically where the spatial gradient is 0^38^. From the results for the transmitted light distributions measured by changing the incident polarization direction *θ* as shown in Fig. 5, it is possible to estimate the in-plane distribution of the liquid crystal molecular orientation. Figure 6(b) shows the in-plane distribution of the liquid crystal molecular orientation within the lens. The out-of-plane vibrational distribution on the surface of glass plate (a) and the orientation direction of the liquid crystal molecules were expressed using a color scale and bars, respectively. The liquid crystal orientation distributions were determined to be *θ*_max_ + 45° by measuring the incident polarization direction *θ*_max_, which then gave the maximum transmitted light intensity at each measurement point shown in Fig. 5. This became possible because the linearly polarized incident light was polarized elliptically, and the maximum transmitted light intensity could then be obtained under crossed Nicols conditions. The lengths of the bars represent the relative inclinations of the liquid crystal molecules from the vertical direction of the liquid crystal layer; these values can be calculated from the ratio of the maximum to the minimum transmitted light intensity at each measurement point when the incident polarization direction is rotated, as shown in Fig. 5, because this ratio increases with the inclination of liquid crystal molecules from the vertical direction due to the birefringence. The inclination of the liquid crystal molecular orientation between the nodal and antinodal positions of the ultrasound flexural vibration was remarkable. This result implying that the change in the molecular orientation of the liquid crystal was induced by the acoustic radiation force acting on the liquid crystal layer because that is expressed as a function of the spatial gradient of the sound pressure amplitude. However, the distribution of the acoustic radiation force to the liquid crystal layer is dependent on the acoustic fields in the glass plate and the liquid crystal layer and these distributions could not be measured directly. The results indicate that the sound pressure distribution in the liquid crystal layer is correlated with the vibration distribution of the glass plate and the concentric resonance mode was generated at the around center of the lens, which resulted in a change in the molecular orientation. In addition, it should be noted that attenuation of the ultrasound in the liquid crystal layer induced the asymmetric molecular orientation in the thickness direction, enabling the variable-focus function (In fact, the ratio of the vibrational amplitude of glass plate (b) to that of glass plate (a) was 0.97). The molecular orientation in the steady state under ultrasound excitation is determined by the balance among the acoustic radiation force, the anchoring force of the alignment film, and the elastic restoring force of the liquid crystal molecules, and this molecular orientation distribution allows the liquid crystal lens to act as a concave lens (Fig. 6(c)). Although this technique can be applied to optical devices with planar aligned liquid crystal, the optical characteristics should be investigated since liquid crystal materials have elastic anisotropy and the speed of sound depends on the propagation direction of acoustic wave^39^.

**Figure 7:**
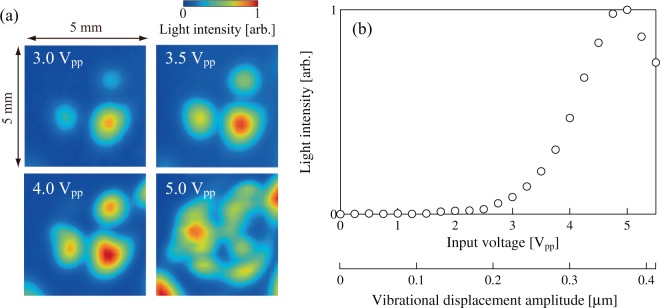
(**a**) Transmitted light intensity distributions through the lens when excited using several different input voltages, and (**b**) relationship between the input voltage to the lens and the transmitted light intensity through the lens at 64.2 kHz.

Figure 7(a,b) show the transmitted light intensity distributions through the lens that were excited using several different input voltages and the relationship between the input voltage and the transmitted light intensity, respectively. In Fig. 7(b), the transmitted light was measured at the position between the node and the antinode of the vibration in the glass plate, where the inclination of the liquid crystal molecular orientation was the greatest ((*x*, *y*) = (1.5 mm, 0 mm) in Fig. 6(b)). There was an input voltage threshold at approximately 2 V_pp_ (an electric power consumption of 2 mW) that induced a change in the transmitted light intensity, and a higher input voltage produced a higher transmitted light intensity stably. Although the maximum transmitted light intensity was saturated when the input voltage increased to more than 5 V_pp_, the light intensity was reduced rapidly within a few seconds after the temporal increase caused by ultrasound excitation. In the case where the input voltage exceeded 5 V_pp_, the liquid crystal layer became turbid and the “dynamic scattering mode” was generated, which also occurs in liquid crystal devices based on use of electric fields^40–42^ (the transmitted light distribution at 5 V_pp_ shown in Fig. 7(a) was measured instantaneously and varied with time). This unstable phenomenon is attributed to turbulence in the liquid crystal layer that is induced by the acoustic radiation force and the secondary torque of the liquid crystal molecules. The tunable range of the focal point largely depends on the birefringence of liquid crystal *Δn*, the lens aperture, and the liquid crystal layer thickness, and thicker liquid crystal layer gives larger tunable range. However, the anchoring force of the liquid crystal molecules to the alignment films decreases as the liquid crystal layer thickness increases, resulting in lower voltage threshold of “dynamic scattering mode”. This fact implies that there is an optimal liquid crystal layer thickness for the tunable lens.

## Conclusions

In this paper, a method to estimate the orientation direction of liquid crystal molecules under ultrasound excitation also the relationship between the ultrasound vibration and the molecular orientation of the liquid crystal were discussed. An ultrasound liquid crystal lens based on use of a piezoelectric ring was fabricated and its optical characteristics were investigated under crossed Nicols conditions. The orientation direction and the inclination in the thickness direction of the liquid crystal layer were determined based on the transmitted light intensity distributions when the incident polarization direction was varied. The molecular orientation was changed axisymmetrically by the acoustic radiation force; the liquid crystal molecules were gradually oriented toward the center of the lens between the node and the antinode of the ultrasound vibration and the vertical orientation of molecules was maintained consistently at the center position that corresponds to the vibrational antinode, thus enabling a variable-focus function.

## References

[1] Mizoguchi, N., Oku, H. & Ishikawa, M. High-speed variable-focus optical system for extended depth of field. *IEEE International Symposium on Industrial Electronics, Seoul*, 1668–1673 (2009).

[2] Liu C (2009). Design and characterization of miniature auto-focusing voice coil motor actuator for cell phone camera applications. IEEE Trans. on Magn.

[3] Oku H, Ishikawa M (2009). High-speed liquid lens with 2 ms response and 80.3 nm root-mean-square wavefront error. Appl. Phys. Lett..

[4] Koyama D, Isago R, Nakamura K (2010). Compact, high-speed variable-focus liquid lens using acoustic radiation force. Opt. Express.

[5] Koyama D, Isago R, Nakamura K (2011). Three-dimensional variable-focus liquid lens using acoustic radiation force. IEEE Trans. Ultrason., Ferroelect., Freq. Contr.

[6] Koyama D, Isago R, Nakamura K (2012). Ultrasonic variable-focus optical lens using viscoelastic material. Appl. Phys. Lett..

[7] Koyama D, Hatanaka M, Nakamura K, Matsukawa M (2012). Ultrasonic optical lens array with variable focal length and pitch. Opt. Lett..

[8] Koyama D, Kashihara Y, Hatanaka M, Nakamura K, Matsukawa M (2016). Movable optical lens array using ultrasonic vibration. Sens. Actuators A, Phys.

[9] Schadt M, Helfrich W (1971). Voltage-dependent optical activity of a twisted nematic crystal. Appl. Phys. Lett..

[10] Schiekel MF, Fahrenschon K (1971). Deformation of nematic liquid crystals with vertical orientation in electrical fields. Appl. Phys. Lett..

[11] Stephen MJ, Straley JP (1974). Physics of liquid crystal. Rev. Mod. Phys..

[12] Sato S (1979). Liquid-crystal lens-cells with variable focal length. Jpn. J. Appl. Phys..

[13] Nose T, Sato S (1989). A liquid crystal microlens obtained with a non-uniform electric field. Liq. Cryst..

[14] Nose, T., Masuda, S. & Sato, S. Optical properties of a liquid crystal microlens with a symmetric electrode structure. Jpn. J. Appl. Phys., **30**(Part 2), 12B, L2110–L2112 (1991).

[15] Wang B, Ye M, Sato S (2006). Liquid crystal lens with focal length variable from negative to positive values. IEEE Photonics Technol. Lett.

[16] Ye M, Wang B, Sato S (2006). Polarization-independent liquid crystal lens with four liquid crystal layers. IEEE Photonics Technol. Lett..

[17] Kawamura M, Nakamura K, Sato S (2013). Liquid-crystal micro-lens array with two-divided and tetragonally hole-patterned electrodes. Opt. Express.

[18] Azuma K (2014). Facile fabrication of transparent and conductive nanowire networks by wet chemical etching with an electrospun nanofiber mask template. Mater. Lett..

[19] Perkowski P (2009). Dielectric spectroscopy of liquid crystals. Theoretical model of ITO electrodes influence on dielectric measurements. Opto-Electron. Rev.

[20] Kawazoe H (1997). P-type electrical conduction in transparent thin films of CuAlO. Nature.

[21] Fujikake H, Sato H, Murashige T (2004). Polymer-stabilized ferroelectric liquid crystal for flexible displays. Displays.

[22] Sato H, Fujikake H, Kikuchi H, Kurita T, Sato F (2005). Rollable ferroelectric liquid crystal devices monostabilized with molecular aligned polymer walls and networks. Liq. Cryst..

[23] Vosgueritchian M, Lipomi DJ, Bao Z (2012). Highly conductive and transparent PEDOT: PSS films with a fluorosurfactant for stretchable and flexible transparent electrodes. Adv. Funct. Mater..

[24] Mailar H, Likins KL, Taylor TR, Fergason JL (1971). Effect of ultrasound on a nematic liquid crystal. Appl. Phys. Lett..

[25] Helfrich W (1972). Orienting action of sound on nematic liquid crystals. Phys. Rev. Lett..

[26] Nagai S, Iizuka K (1974). On the effect of ultrasound to nematic liquid crystal. Jpn. J. Appl. Phys..

[27] Taniguchi S (2016). Control of liquid crystal molecular orientation using ultrasound vibration. Appl. Phys. Lett..

[28] Shimizu Y (2017). Periodic pattern of liquid crystal molecular orientation induced by ultrasound vibrations. Appl. Phys. Lett..

[29] Shimizu Y (2018). Ultrasound liquid crystal lens. Appl. Phys. Lett..

[30] Scheffer TJ, Nehring J (1977). Accurate determination of liquid-crystal tilt bias angles. J. Appl. Phys..

[31] Qin J, Iwami K, Umeda N (2008). Direct evaluation of anchoring effects and vertical orientation profiling of liquid crystal films by Near-Field Birefringence Measurement. Appl. Phys. Express..

[32] Tadokoro, T., Saiki, T. & Toriumi, H. Design and implementation of Near-Field Scanning Optical Microscope for observation of interfacial liquid crystal orientation. *Jpn. J. Appl. Phys*. **41**(Part 2), 2A, L152–L154 (2002).

[33] Tadokoro, T., Saiki, T. & Toriumi, H. Two-dimensional analysis of liquid crystal orientation at in-plane switching substrate surface using a Near-Field Scanning Optical Microscope. Jpn. J. Appl. Phys., 42(Part 2), 1A/B, L57–L59 (2003).

[34] Hertz G, Mende H (1939). Der Schallstrahlungsdruck in Flüssigkeiten. Z. Phys.

[35] Rozenberg LD, Makarov LO (1958). On the causes of ultrasonic distension of liquid surfaces. Sov. Phys. Dokl.

[36] Chu B, Apfel E (1982). Acoustic radiation pressure produced by a beam of sound. J. Acoust. Soc. Am..

[37] Rayleigh, J. W. S., The Theory of Sound, Vol. 2, 297 (Dover, 1945).

[38] Koyama D, Nakamura K (2010). Noncontact ultrasonic transportation of small objects over long distances in air using a bending vibrator and a reflector. IEEE Trans. Ultrason., Ferroelect., Freq. Contr.

[39] Natale GG (1978). The contribution of ultrasonic measurements to the study of liquid crystals. I. Nematics. J. Acoust. Soc. Am..

[40] Heilmeier GH, Zanoni LA, Barton LA (1968). Dynamic scattering: A new electrooptic effect in certain classes of nematic liquid crystals. Proc. IEEE.

[41] Kessler LW, Sawyer SP (1970). Ultrasonic stimulation of optical scattering in nematic liquid crystals. Appl. Phys. Lett..

[42] Serak SV (2016). High contrast switching of transmission due to electrohydrodynamic effect in stacked thin systems of liquid crystals. Appl. Opt..

